# Identification and immunological characterization of cuproptosis-related molecular clusters in Alzheimer's disease

**DOI:** 10.3389/fnagi.2022.932676

**Published:** 2022-07-28

**Authors:** Yongxing Lai, Chunjin Lin, Xing Lin, Lijuan Wu, Yinan Zhao, Fan Lin

**Affiliations:** ^1^Department of Geriatric Medicine, Shengli Clinical Medical College of Fujian Medical University, Fuzhou, China; ^2^Fujian Provincial Center for Geriatrics, Fujian Provincial Hospital, Fuzhou, China

**Keywords:** Alzheimer's disease, cuproptosis, molecular clusters, immune infiltration, machine learning, prediction model

## Abstract

**Introduction:**

Alzheimer's disease is the most common dementia with clinical and pathological heterogeneity. Cuproptosis is a recently reported form of cell death, which appears to result in the progression of various diseases. Therefore, our study aimed to explore cuproptosis-related molecular clusters in Alzheimer's disease and construct a prediction model.

**Methods:**

Based on the GSE33000 dataset, we analyzed the expression profiles of cuproptosis regulators and immune characteristics in Alzheimer's disease. Using 310 Alzheimer's disease samples, we explored the molecular clusters based on cuproptosis-related genes, along with the related immune cell infiltration. Cluster-specific differentially expressed genes were identified using the WGCNA algorithm. Subsequently, the optimal machine model was chosen by comparing the performance of the random forest model, support vector machine model, generalized linear model, and eXtreme Gradient Boosting. Nomogram, calibration curve, decision curve analysis, and three external datasets were applied for validating the predictive efficiency.

**Results:**

The dysregulated cuproptosis-related genes and activated immune responses were determined between Alzheimer's disease and non-Alzheimer's disease controls. Two cuproptosis-related molecular clusters were defined in Alzheimer's disease. Analysis of immune infiltration suggested the significant heterogeneity of immunity between distinct clusters. Cluster2 was characterized by elevated immune scores and relatively higher levels of immune infiltration. Functional analysis showed that cluster-specific differentially expressed genes in Cluster2 were closely related to various immune responses. The Random forest machine model presented the best discriminative performance with relatively lower residual and root mean square error, and a higher area under the curve (AUC = 0.9829). A final 5-gene-based random forest model was constructed, exhibiting satisfactory performance in two external validation datasets (AUC = 0.8529 and 0.8333). The nomogram, calibration curve, and decision curve analysis also demonstrated the accuracy to predict Alzheimer's disease subtypes. Further analysis revealed that these five model-related genes were significantly associated with the Aβ-42 levels and β-secretase activity.

**Conclusion:**

Our study systematically illustrated the complicated relationship between cuproptosis and Alzheimer's disease, and developed a promising prediction model to evaluate the risk of cuproptosis subtypes and the pathological outcome of Alzheimer's disease patients.

## Introduction

Alzheimer's disease (AD) is the most common form of age-related neurodegenerative disease. It is reported that approximately more than 42.3 million people worldwide suffer from progressive cognitive impairment caused by AD (Ambrogio et al., 2019), and data from epidemiological analyses suggest that the number of people with AD will be more than twice the current number in 2060 (Matthews et al., 2019). As the disease progresses, AD patients may experience varying degrees of cognitive and memory insult, such as language, visuospatial, motor, and executive function deficits (McKhann et al., 2011). The increasing number of AD patients, therefore, places a huge burden on families and society. Unfortunately, due to the clinical heterogeneity of AD and the complexity of pathological types, satisfactory treatment for AD was lacking and no effective strategy was proven to prevent the occurrence of AD (Nandigam, 2008; Rahimi and Kovacs, 2014). Recently, a growing number of biomarkers are associated with AD, yet a single dataset or relatively small sample sizes may make these results unconvincing (Zheng et al., 2018; Liu et al., 2022). In addition, the efficacy of biomarker-based univariate prediction models has also been challenged (Jack et al., 2014). Therefore, further accurate identification of molecular subtypes of AD at the molecular level and establishing a multivariate predictive model would be of great clinical importance.

As cofactors for enzymes, the maintenance of copper ions (Cu^2+^), copper homeostasis, mainly depends on the regulation of mitochondria (Baker et al., 2017). Copper exists mainly in the form of cytochrome C oxidase (COX) and superoxide dismutase (SOD1) in mitochondria, thus acting as a vital regulator in the tricarboxylic acid cycle (TCA) and eventually playing a critical role in various biological processes including redox balance, iron utilization, oxidative phosphorylation, and cell growth (Soto et al., 2012; Dennerlein and Rehling, 2015). However, dysregulation of copper homeostasis has been proven to be associated with neurodegenerative diseases (Gromadzka et al., 2020). Cuproptosis is a recently discovered novel form of cell death that differs from other oxidative stress-regulated deaths such as pyroptosis, ferroptosis, and necroptosis. It is reported that mitochondrial stress characterized by over-accumulation of lipoylated mitochondrial enzymes and the depletion of Fe-S cluster proteins are the primary mechanisms causing cuproptosis (Oliveri, 2022; Wang et al., 2022b). Cu^2+^ entering the cell can be reduced to Cu^+^ by FDX1, which in turn promotes lipid acylation of mitochondrial proteins and overproduction of key enzymes associated with the mitochondrial tricarboxylic acid (TCA) cycle (DBT, GCSH, DLST, and DLAT). In addition, the instability of Fe-S cluster proteins is also closely related to FDX1. Moreover, critical genes implicated in the Cu^2+^ transport, such as SLC31A1 and ATP7B, may play a vital role in regulating the occurrence of cuproptosis. Furthermore, it was found that the inhibition of mitochondrial pyruvate carrier and electron transport chain activity could alleviate the damage caused by cuproptosis (Cobine and Brady, 2022; Tang et al., 2022; Tsvetkov et al., 2022). In addition, an increasing number of studies demonstrate that mitochondria dysfunction-induced deficiency of energy metabolism and oxidative stress may be the critical pathogenesis involved in AD progression (Chen and Zhong, 2014; Murphy and Hartley, 2018; Tang et al., 2019). Therefore, it would be reasonable to infer that cuproptosis is closely associated with the development of AD. However, the potential regulatory mechanisms of cuproptosis in AD remain unknown and require further exploration. Therefore, further illustrating the molecular characteristics of cuproptosis-related genes (CRGs) may be able to explain the cause of heterogeneity in AD.

In the present study, we systematically examined the differentially expressed CRGs and immune characteristics for the first time between normal and AD individuals. Based on the 13 CRGs expression landscapes, we classified 310 AD patients into two cuproptosis-related clusters, and immune cell differences between the two clusters were further evaluated. Subsequently, cluster-specific DEGs were identified using the WGCNA algorithm, and the enriched biological functions and pathways were elucidated based on cluster-specific DEGs. In addition, a prediction model for disclosing patients with different molecular clusters was established by comparing multiple machine learning algorithms. The nomogram, calibration curve, decision curve analysis (DCA), and two external datasets were used to validate the performance of the predictive model. Finally, we further investigated the correlation between model-related genes with β-secretase activity and Aβ-42 levels in another external AD cohort, thus providing novel insights into the prediction of AD clusters and risk.

## Materials

### Data acquisition and pre-processing

Four microarray datasets (GSE33000, GSE5281, GSE122063, and GSE106241) related to AD were obtained from the GEO website database (GEO, www.ncbi.nlm.nih.gov/geo) using the “GEOquery” R package (version 2.60) (Davis and Meltzer, 2007). The GSE33000 dataset (GPL4372 platform) including 157 healthy (age: 22 to 106 years) and 310 AD (age: 53 to 100 years) cortex tissue samples were selected for further analysis. The GSE5281 dataset (GPL570 platform), which included brain tissues from 74 normal (age: 68 to 97 years) subjects and 87 AD (age: 63 to 102 years) samples; the GSE122063 dataset (GPL16699 platform), which included cortex tissues from 44 normal (age: 60 to 91 years) samples and 56 AD (age: 63–91 years) samples, and the GSE106241 dataset (GPL24170 platform), which included cortex tissues from 40 AD (age: 50–100 years) samples, were selected for validation analysis. The raw gene expression profiles of these GEO datasets were processed and normalized using the Robust Multiarray Average (RMA) method (“affy” R package, version 1.70.0).

### Evaluating the immune cell infiltration

The CIBERSORT algorithm (https:/cibersort.stanford.edu/) and LM22 signature matrix were applied for estimating the relative abundances of 22 types of immune cells in each sample based on the proceeded gene expression data. CIBERSORT uses Monte Carlo sampling to obtain an inverse fold product *p*-value for each sample. Only samples with *p*-values <0.05 were considered to be accurate immune cell fractions. The sum of the 22 immune cells proportions in each sample was 1 (Newman et al., 2015).

### Correlation analysis between CRGs and infiltrated immune cells

To further demonstrate the association between CRGs and AD-related immune cell properties, we analyzed the correlation coefficients between the CRGs expression and the relative percentage of immune cells. According to the spearman correlation coefficient, *p*-values below 0.05 represented a significant correlation. Finally, the results were exhibited using the “corrplot” R package (version 0.92).

### Unsupervised clustering of AD patients

Initially, a total of 13 CRGs were obtained according to the previous report by Tsvetkov et al. (2022). Based on 13 CRGs expression profiles, we applied the unsupervised clustering analysis (“ConsensusClusterPlus” R package, version 2.60) (Wilkerson and Hayes, 2010) classifying the 310 AD samples into different clusters by using the k-means algorithm with 1,000 iterations. We chose a maximum subtype number k (k = 6) and the optimal cluster number was comprehensively evaluated based on the cumulative distribution function (CDF) curve, consensus matrix, and consistent cluster score (>0.9).

### Gene set variation analysis (GSVA) analysis

GSVA enrichment analysis was conducted to elucidate the differences in enriched gene sets between different CRGs clusters using the R package of “GSVA” (version 2.11). The “c2.cp.kegg.v7.4.symbols” and “c5.go.bp.v7.5.1.symbols” files were obtained from the MSigDB website database for further GSVA analysis. The “limma” R package (version 3.52.1) was utilized to identify the differentially expressed pathways and biological functions by comparing GSVA scores between different CRGs clusters. The |t value of GSVA score| more than 2 was considered as significantly altered.

### Weighted gene co-expression network analysis (WGCNA)

WGCNA was performed to identify co-expression modules using the R package of “WGCNA” (version 1,70.3) (Langfelder and Horvath, 2008). The top 25% of genes with the highest variance were applied for subsequent WGCNA analyses to guarantee the accuracy of quality results. We selected an optimal soft power to construct a weighted adjacency matrix and further transformed it into a topological overlap matrix (TOM). Modules were obtained using the TOM dissimilarity measure (1-TOM) based on the hierarchical clustering tree algorithm when the minimum module size was set to 100. Each module was assigned a random color. Module eigengene represented the global gene expression profiles in each module. The relationship between modules and disease status was exhibited by the modular significance (MS). Gene significance (GS) was described as the correlation between a gene with clinical phenotype.

### Construction of predictive model based on multiple machine learning methods

Based on two different CRGs clusters, we applied the “caret” R packages (version 6.0.91) for establishing machine learning models including random forest model (RF), support vector machine model (SVM), generalized linear model (GLM), and eXtreme Gradient Boosting (XGB). RF is an ensemble machine learning approach utilizing various independent decision trees for the prediction of classification or regression (Rigatti, 2017). SVM algorithm enables to generate a hyperplane in the characteristic space with a maximum margin to distinguish between positive and negative instances (Gold and Sollich, 2003). GLM, an extension of multiple linear regression models, could flexibly evaluate the relationship between normally distributed dependent features and categorical or continuous independent features (Nelder and Wedderburn, 1972). XGB is an ensemble of boosted trees based on gradient boosting, which can make a careful comparison between classification error and model complexity (Chen et al., 2015). The distinct clusters were considered as the response variable, and the cluster-specific DEGs were selected as explanatory variables. The 310 AD samples were randomly classified into a training set (70%, *N* = 217) and a validation set (30%, *N* = 93). The caret package automatically tuned the parameters in these models by grid search, and all of these machine learning models were performed with default parameters and assessed via 5-fold cross-validation. The “DALEX” package (version 2.4.0) was carried out to interpret the aforementioned four machine learning models and visualize the residual distribution and feature importance among these machine learning models. The “pROC” R package (version 1.18.0) was performed to visualize the area under ROC curves. Consequently, the optimal machine learning model was determined and the top five important variables were considered as the key predictive genes associated with AD. Finally, The ROC curves analysis were performed in GSE5281 and GSE122063 datasets to verify the diagnostic value of the diagnostic model.

### Construction and validation of a nomogram model

A nomogram model was established to evaluate the occurrence of AD clusters using the “rms” R package (version 6.2.0). Each predictor has a corresponding score, and the “total score” represents the sum of the scores of the above predictors. The calibration curve and DCA were utilized to estimate the predictive power of the nomogram model.

### Independent validation analysis

Two external brain tissue datasets, GSE5281 and GSE122063, were applied for validating the ability of the prediction model to distinguish AD from non-AD controls by using the ROC analyses. ROC curves were visualized using the “pROC” R package. In addition, we performed the spearman correlation analysis to explore the associations between prediction model-related genes with Aβ-42 levels and β-secretase activity. A value of *p* < 0.05 was considered as statistically significant.

## Results

### Dysregulation of cuproptosis regulators and activation of the immune responses in AD patients

**Figure 1:**
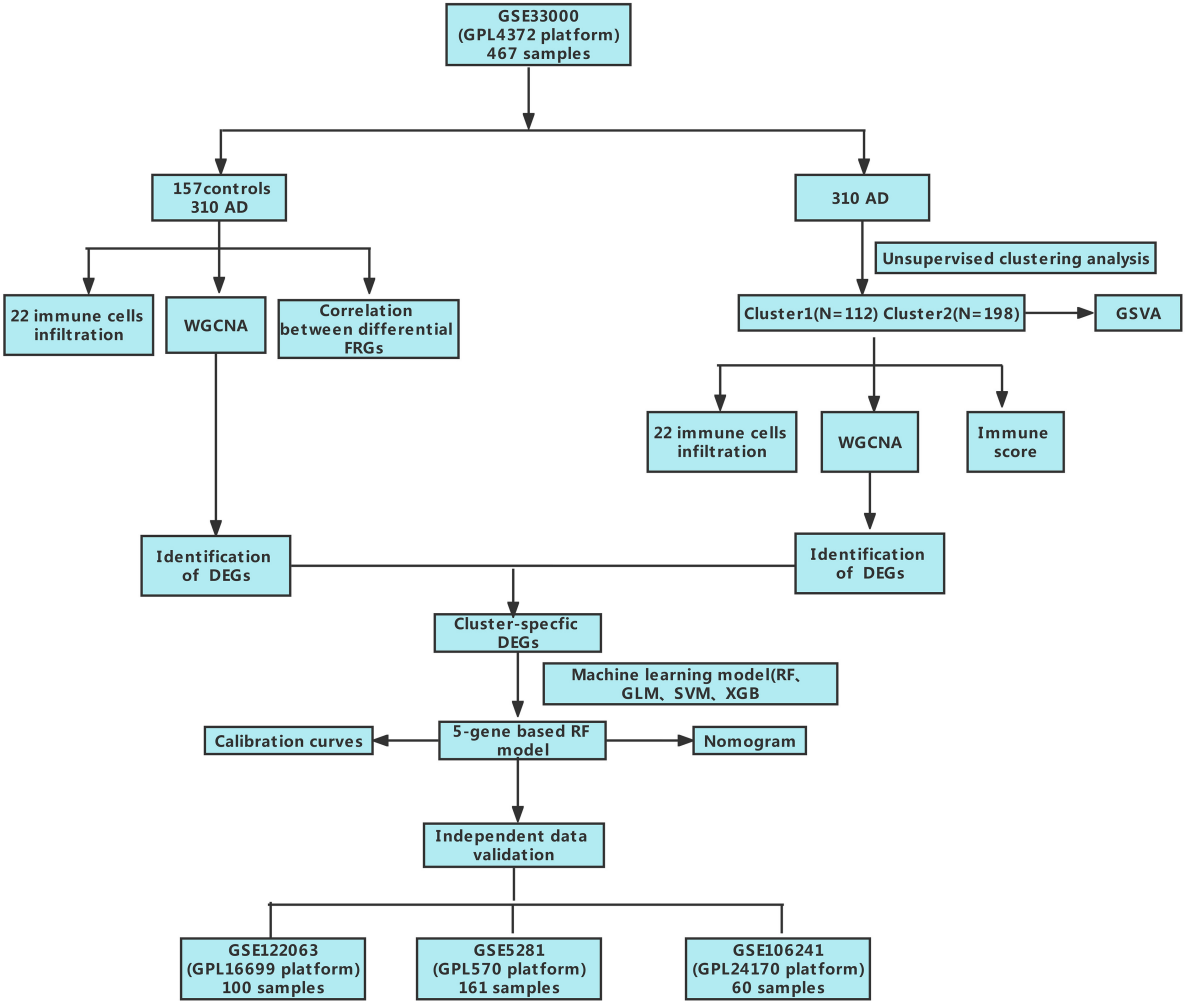
The study flow chart.

**Figure 2:**
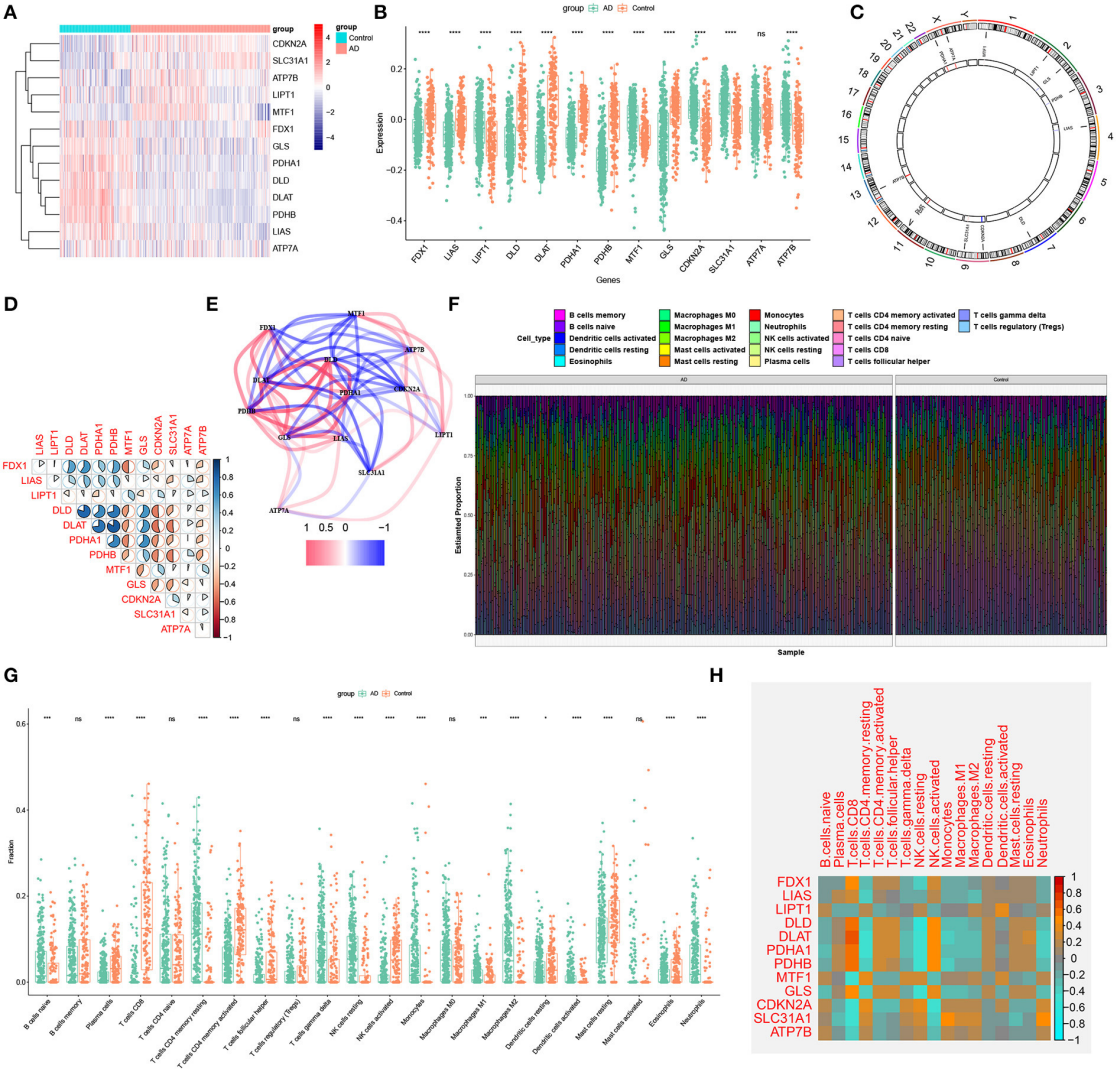
Identification of dysregulated CRGs in AD. **(A)** The expression patterns of 13 CRGs were presented in the heatmap. **(B)** Boxplots showed the expression of 13 CRGs between AD and non-AD controls. ****p < 0.0001, ns, no significance. **(C)** The location of 13 CRGs on chromosomes. **(D)** Correlation analysis of 12 differentially expressed CRGs. Blue and Red colors represent positive and negative correlations, respectively. The correlation coefficients were marked with the area of the pie chart. **(E)** Gene relationship network diagram of 12 differentially expressed CRGs. **(F)** The relative abundances of 22 infiltrated immune cells between AD and non-AD controls. **(G)** Boxplots showed the differences in immune infiltrating between AD and non-AD controls. *p < 0.05, ***p < 0.001, ****p < 0.0001, ns, no significance. **(H)** correlation analysis between 12 differentially expressed CRGs and infiltrated immune cells.

To clarify the biological functions of cuproptosis regulators in the occurrence and progression of AD, we first systematically evaluated the expression profiles of 13 CRGs between AD and non-AD controls using the GSE33000 dataset. A detailed flow chart of the study process was exhibited in Figure 1. A total of 12 CRGs were determined as the differentially expressed cuproptosis genes. Among them, the expression levels of CDKN2A, SLC31A1, ATP7B, LIPT1, and MTF1 were higher, whereas FDX1, GLS, PDHA1, DLD, DLAT, PDHB, and LIAS gene expression levels were largely lower in AD cortex tissues than that in non-AD controls (Figures 2A–C). Subsequently, we performed the correlation analysis between these differentially expressed CRGs to explore whether cuproptosis regulators functioned essentially in the progression of AD. Surprisingly, some cuproptosis modulators, such as PDHB and DLAT, presented a strong synergistic effect (coefficient = 0.83). Simultaneously, CDKN2A and DLAT exhibited an apparent antagonistic action (coefficient = 0.54). In addition, we further investigated the correlation patterns of these CRGs and found both DLAT and DLD were significantly correlated with other regulators (Figure 2D). The gene relationship network diagram further demonstrated the closeness of the relationship among these differentially expressed CRGs (Figure 2E).

To elucidate whether there is variation in the immune system between the AD and non-AD controls, immune infiltration analysis was performed to show a difference in the proportions of 22 infiltrated immune cell types between AD and non-AD control subjects based on the CIBERSORT algorithm (Figure 2F). The results revealed that AD patients presented higher infiltration levels of naïve B cells, resting memory CD4^+^ T cells, gamma delta T cells, resting NK cells, Monocytes, M1 macrophages, M2 macrophages, activated dendritic cells, and neutrophils (Figure 2G), suggesting that the alternations in the immune system may be a major cause for the occurrence of AD. Meanwhile, correlation analysis results indicated that both resting NK cells and CD8^+^ T cells were correlated with cuproptosis modulators (Figure 2H). These results suggested that CRGs may be the critical factors involved in regulating the molecular and immune infiltration status of AD patients.

### Identification of cuproptosis clusters in AD

**Figure 3:**
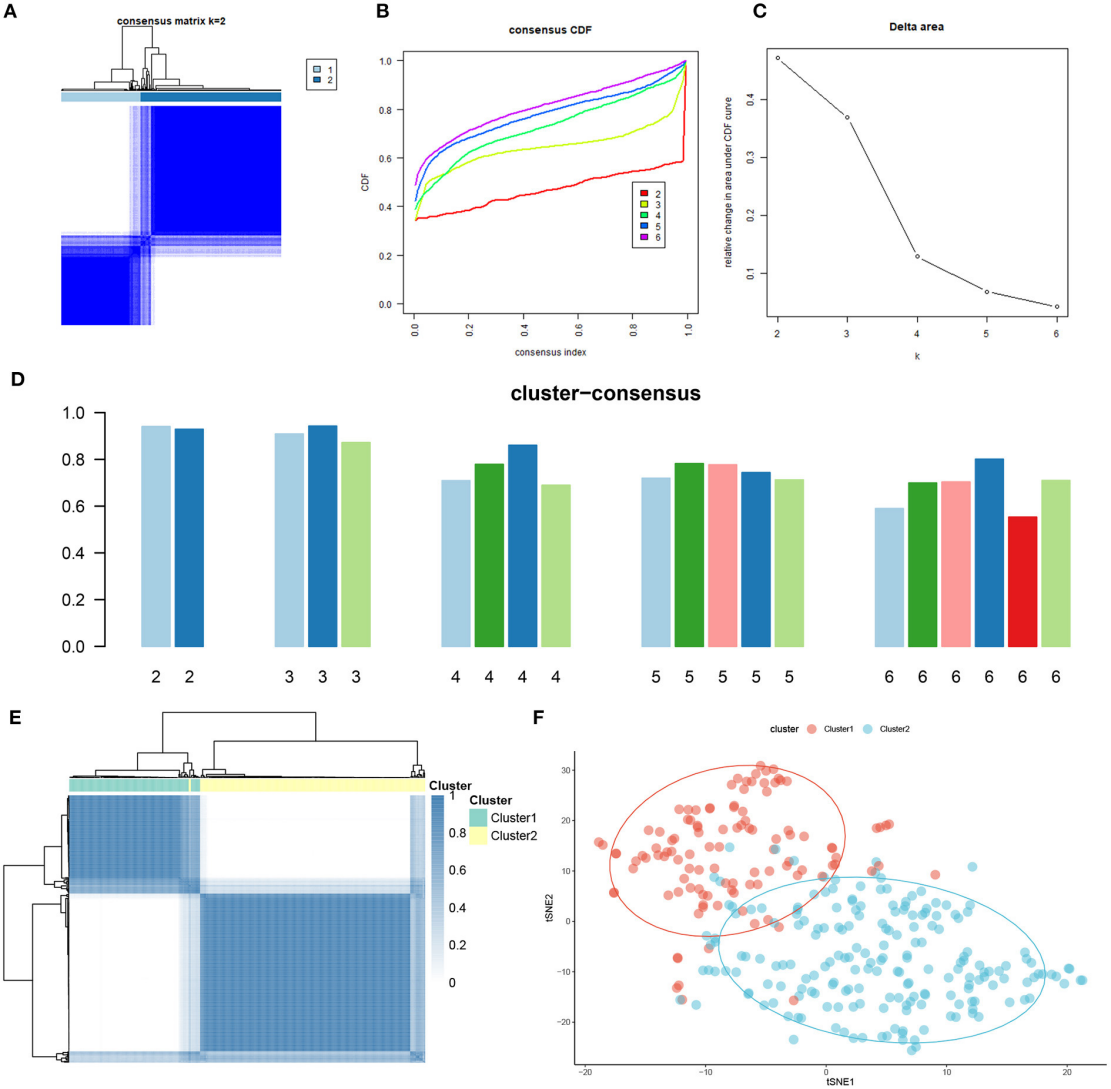
Identification of cuproptosis-related molecular clusters in AD. **(A)** Consensus clustering matrix when k = 2. **(B–E)** Representative cumulative distribution function (CDF) curves **(B)**, CDF delta area curves **(C)**, the score of consensus clustering **(D)**, and heatmap of non-negative matrix **(E)**. **(F)** t-SNE visualizes the distribution of two subtypes.

To elucidate the cuproptosis-related expression patterns in ASD, we grouped the 310 AD samples based on the expression profiles of 13 CRGs using a consensus clustering algorithm. The cluster numbers were most stable when the k value was set to two (k = 2), and the CDF curves fluctuated within a minimum range at a consensus index of 0.2 to 0.6 (Figures 3A,B). When k = 2 to 6, the area under the CDF curves exhibited the difference between the two CDF curves (k and k-1) (Figure 3C). Furthermore, the consistency score of each subtype was >0.9 only when k = 2 (Figure 3D). Conjoined with the heatmap of the consensus matrix, we finally grouped 310 AD patients into two clusters, including Cluster1 (*n* = 112) and Cluster2 (*n* = 198) (Figure 3E). The results of the t-Distributed Stochastic Neighbor Embedding (tSNE) analysis demonstrated that there was a significant difference between these two clusters (Figure 3F).

### Differentiation of cuproptosis regulators and immune infiltration characteristics between cuproptosis clusters

**Figure 4:**
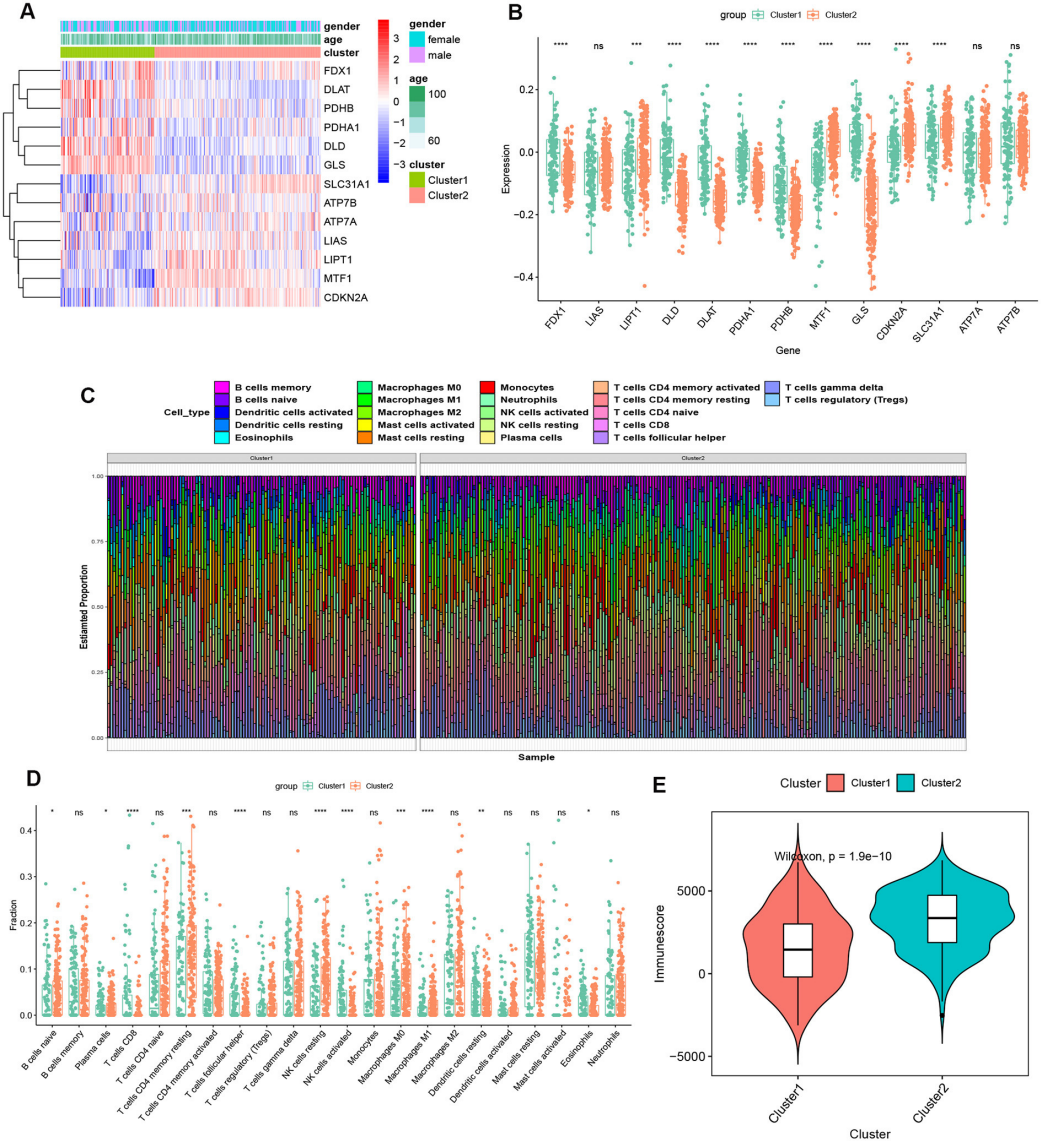
Identification of molecular and immune characteristics between the two cuproptosis clusters. **(A)** Clinical features and expression patterns of 13 CRGs between two cuproptosis clusters were presented in the heatmap. **(B)** Boxplots showed the expression of 13 CRGs between two cuproptosis clusters. ***p < 0.001, ****p < 0.0001, ns, no significance. **(C)** the relative abundances of 22 infiltrated immune cells between two cuproptosis clusters. **(D)** Boxplots showed the differences in immune infiltrating between two cuproptosis clusters. *p < 0.05, **p < 0.01 ***p < 0.001, ****p < 0.0001, ns, no significance. **(E)** Boxplots showed the estimated immune score between the two cuproptosis subtypes.

To explore the molecular characteristics between clusters, we first comprehensively assessed the expression differences of 13 CRGs between Cluster1 and Cluster2. Distinct CRGs expression landscapes were observed between the two cuproptosis patterns (Figure 4A). Cuproptosis Cluster1 revealed high expression levels of FDX1, DLD, DLAT, PDHA1, PDHB, and GLS, while cuproptosis Cluster2 was characterized by enhanced expressions of LIPT1, MTF1, CDKN2A, and SLC31A1 (Figure 4B). Moreover, the results of immune infiltration analysis showed that an altered immune microenvironment was presented between cuproptosis Cluster1 and Cluster2 (Figure 4C). Cluster1 exhibited higher proportions of CD8+ T cells, follicular helper T cells, activated NK cells, resting dendritic cells, and eosinophils, whereas the abundance of naïve B cells, Plasma cells, naïve CD4+ T cells, resting memory CD4+ T cells, resting NK cells, Monocytes, M0 macrophages, and M1 macrophages were relatively greater in Cluster2 (Figure 4D). Consistently, Cluster2 also presented elevated immune scores (Figure 4E), which revealed that cuproptosis Cluster2 might possess a more dominant level of immune infiltration.

### Gene modules screening and co-expression network construction

**Figure 5:**
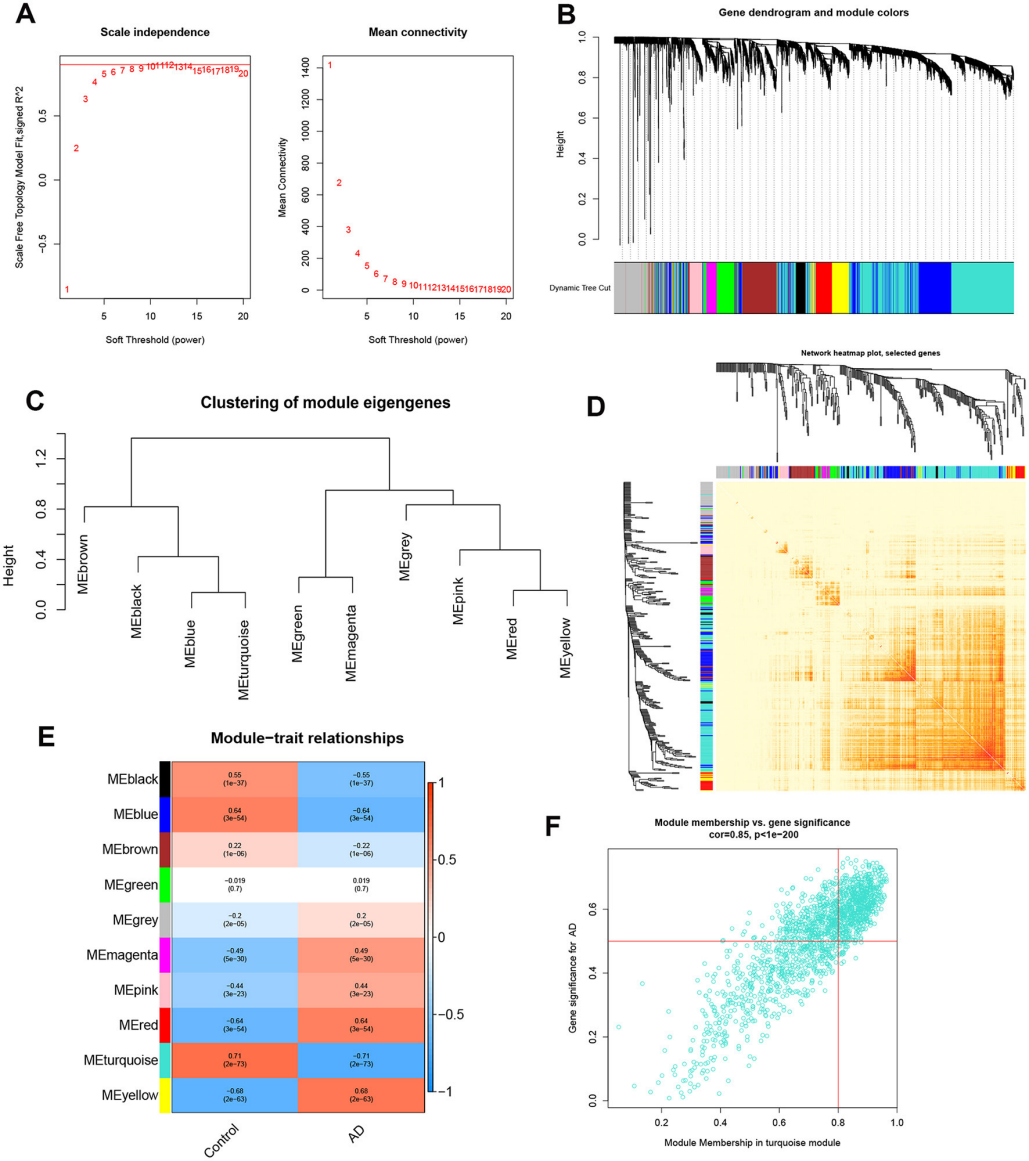
Co-expression network of differentially expressed genes in AD. **(A)** The selection of soft threshold power. **(B)** Cluster tree dendrogram of co-expression modules. Different colors represent distinct co-expression modules. **(C)** Representative of clustering of module eigengenes. **(D)** Representative heatmap of the correlations among 10 modules. **(E)** Correlation analysis between module eigengenes and clinical status. Each row represents a module; each column represents a clinical status. **(F)** Scatter plot between module membership in turquoise module and the gene significance for AD.

To identify the key gene modules associated with AD, we utilized the WGCNA algorithm to establish a co-expression network and modules for the normal and AD subjects. We calculated the variance of each gene expression in GSE33000 and then selected the top 25% genes with the highest variance to further analysis. Co-expressed gene modules were identified when the value of soft power was set to 9 and the scale-free *R*^2^ was equal to 0.9 (Figure 5A). A total of 10 distinct co-expression modules with different colors were acquired using the dynamic cutting algorithm and the heatmap of the topological overlap matrix (TOM) was also presented (Figures 5B–D). Subsequently, these genes in the 10 color modules were continuously applied for analyzing the similarity and adjacency of module-clinical features (Control and AD) co-expression. Finally, the turquoise module exhibited the strongest relationship with AD, which included 1,609 genes (Figure 5E). Moreover, we observed a positive association between the turquoise module and module-related genes (Figure 5F).

**Figure 6:**
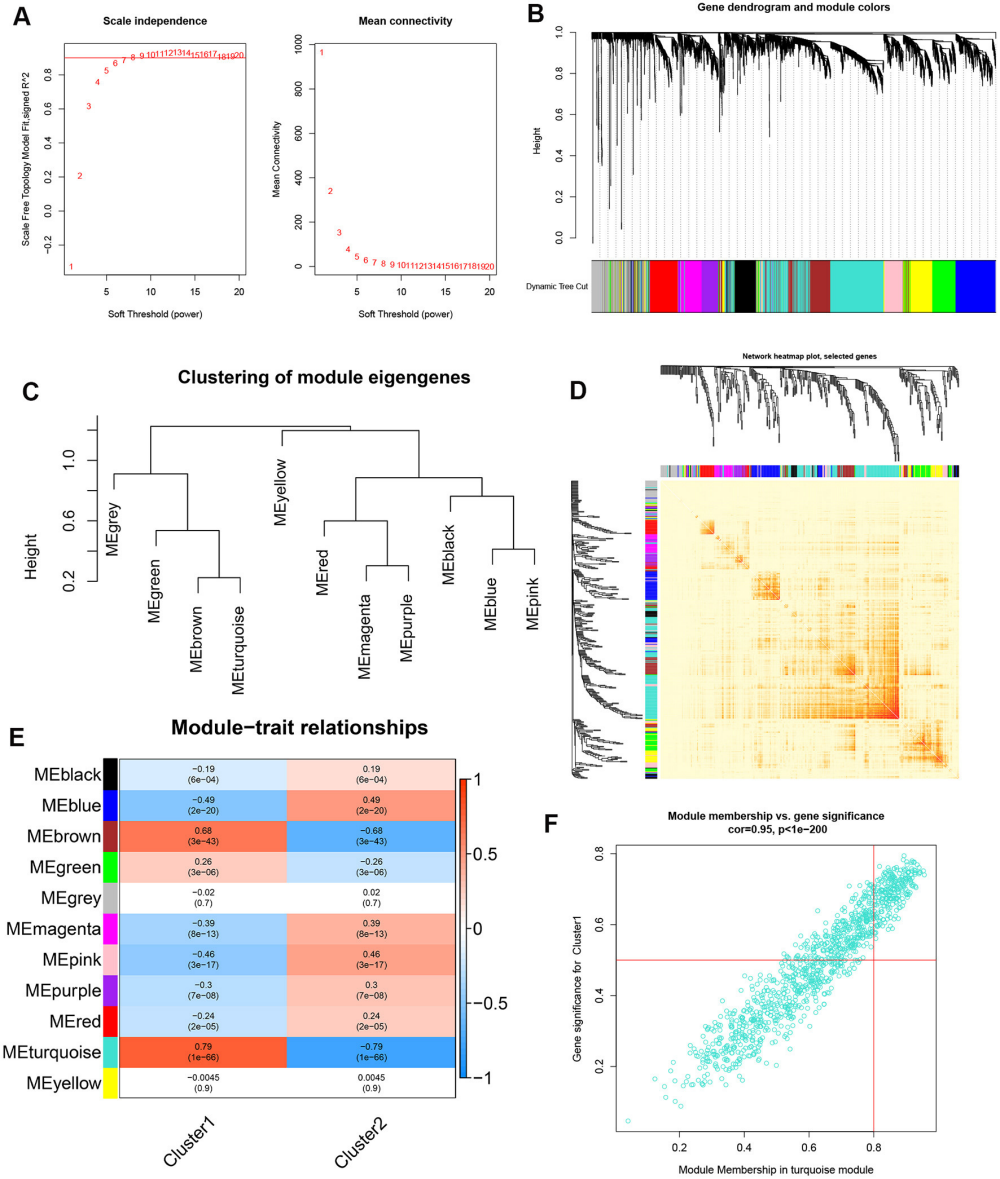
Co-expression network of differentially expressed genes between the two cuproptosis clusters. **(A)** The selection of soft threshold power. **(B)** Cluster tree dendrogram of co-expression modules. Different colors represent distinct co-expression modules. **(C)** Representative of clustering of module eigengenes. **(D)** Representative heatmap of the correlations among 11 modules. **(E)** Correlation analysis between module eigengenes and clinical status. Each row represents a module; each column represents a clinical status. **(F)** Scatter plot between module membership in turquoise module and the gene significance for Cluster1.

In addition, we also analyzed the critical gene modules closely related to cuproptosis clusters using the WGCNA algorithm. We screened β = 7 and *R*^2^ = 0.9 as the most suitable soft threshold parameters to construct a scale-free network (Figure 6A). Specifically, 11 modules containing 4,422 genes were determined as significant modules and the heatmap portrayed the TOM of all module-related genes (Figures 6B–D). Module-clinical features (Cluster1 and Cluster2) relationship analysis demonstrated the high correlation between the turquoise module (1,115 genes) and AD clusters (Figure 6E). The correlation analysis suggested that turquoise module genes had a significant relationship with the selected module (Figure 6F).

### Identification of cluster-specific DEGs and functional annotation

**Figure 7:**
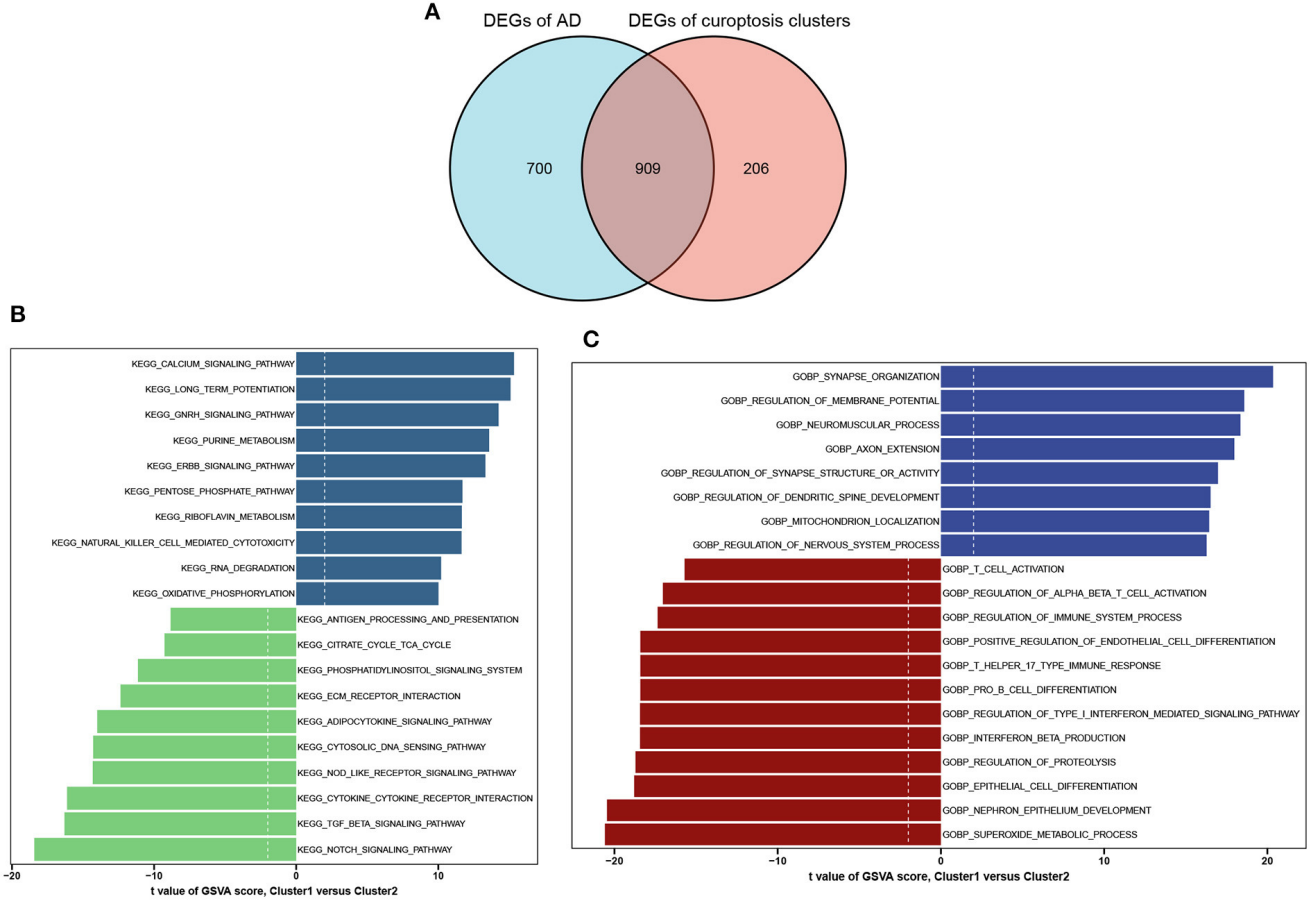
Identification of cluster-specific DEGs and biological characteristics between two cuproptosis clusters. **(A)** The intersections between module-related genes of cuproptosis clusters and module-related genes in the GSE33000 dataset. **(B)** Differences in hallmark pathway activities between Cluster1 and Cluster2 samples ranked by *t*-value of GSVA method. **(C)** Differences in biological functions between Cluster1 and Cluster2 samples ranked by *t*-value of GSVA method.

A total of 909 cluster-specific DEGs were identified by analyzing the intersections between module-related genes of cuproptosis clusters and module-related genes of AD and non-AD individuals (Figure 7A). The GSVA analysis was utilized to further explore the functional differences associated with cluster-specific DEGs between the two clusters. The results indicated that oxidative phosphorylation, RNA degradation, long-time potentiation, and metabolism signaling activity were reinforced in Cluster1, while the TCA cycle, immune responses, cytokine receptor, TGF-β, and Notch signaling activity were upregulated in Cluster2 (Figure 7B). Otherwise, functional enrichment results revealed that Cluster1 was remarkably related to the regulation of synapse and axon outgrowth, the development of the dendritic spine development, and mitochondrial localization. However, immune-related pathways, such as T-cell activation, B-cell differentiation, and beta-interferon production, were enriched in Cluster2 (Figure 7C). Thus, we hypothesized that Cluster2 may be involved in various immune responses.

### Construction and assessment of machine learning models

**Figure 8:**
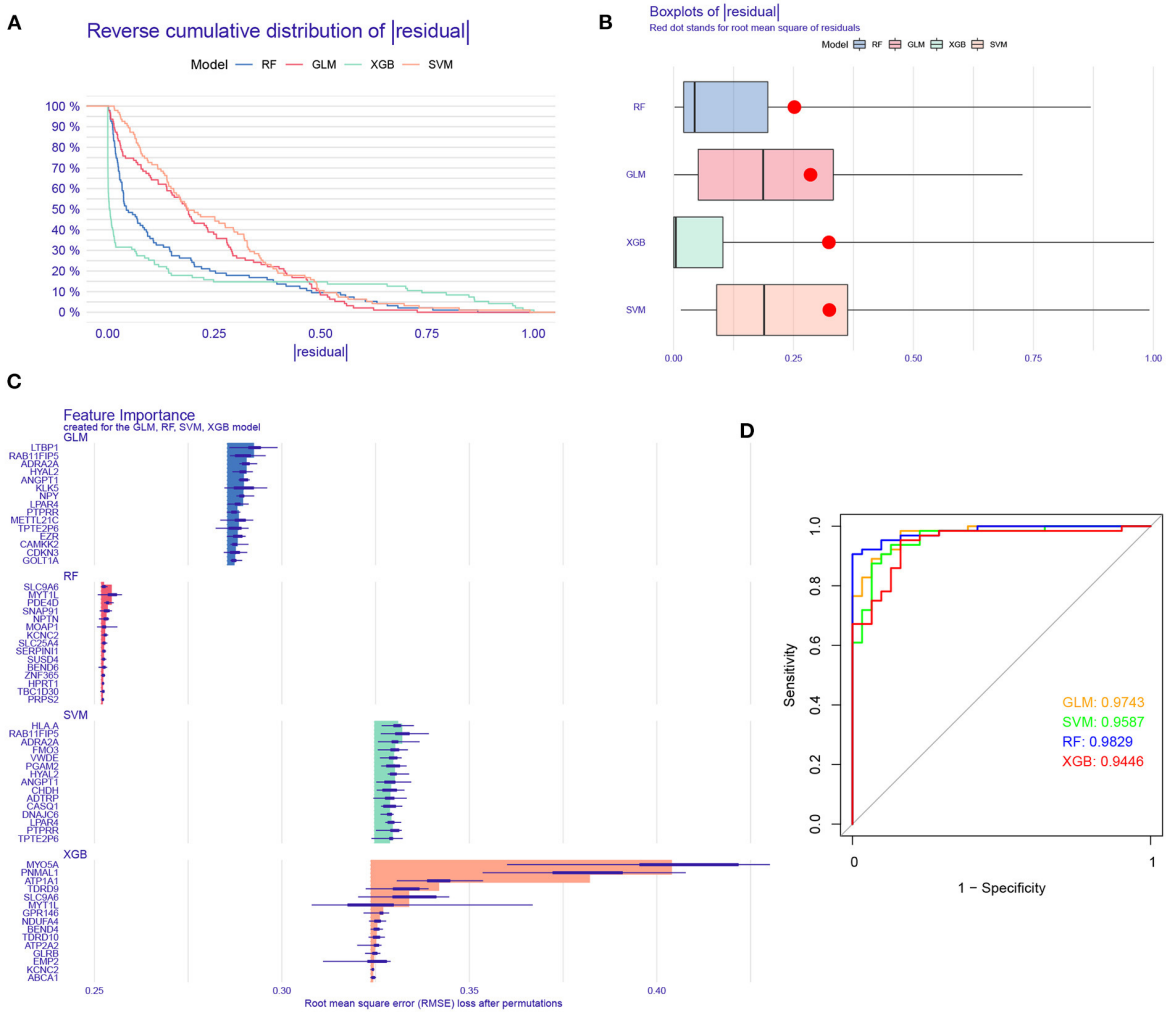
Construction and evaluation of RF, SVM, GLM, and XGB machine models. **(A)** Cumulative residual distribution of each machine learning model. **(B)** Boxplots showed the residuals of each machine learning model. Red dot represented the root mean square of residuals (RMSE). **(C)** The important features in RF, SVM, GLM, and XGB machine models. **(D)** ROC analysis of four machine learning models based on 5-fold cross-validation in the testing cohort.

To further identify subtype-specific genes with high diagnostic value, we established four proven machine learning models [random forest model (RF), support vector machine model (SVM), generalized linear model (GLM), and eXtreme Gradient Boosting (XGB)] based on the expression profiles of 909 cluster-specific DEGs in the AD training cohort. The “DALEX” package was applied for explaining the four models and plotting the residual distribution of each model in the test set. RF and GLM machine learning models presented a relatively lower residual (Figures 8A,B). Subsequently, the top 15 important feature variables of each model were ranked according to the root mean square error (RMSE) (Figure 8C). Moreover, we evaluated the discriminative performance of the four machine learning algorithms in the testing set by calculating receiver operating characteristic (ROC) curves based on 5-fold cross-validation. The RF machine learning model displayed the highest area under the ROC curve (AUC) (GLM, AUC = 0.9743; SVM, AUC = 0.9587; RF, AUC =0.9829; XGB, AUC = 0.9446, Figure 8D). Overall, combined with these results, the RF model was demonstrated to best distinguish patients with different clusters. Finally, the top five most important variables (MYT1L, PDE4D, SNAP91, NPTN, and KCNC2) from the RF model were selected as predictor genes for further analysis.

**Figure 9:**
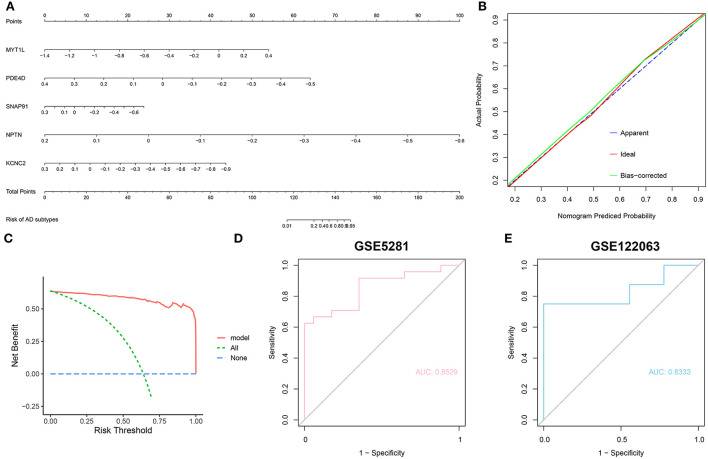
Validation of the 5-gene-based RF model. **(A)** Construction of a nomogram for predicting the risk of AD clusters based on the 5-gene-based RF model. **(B,C)** Construction of calibration curve **(B)** and DCA **(C)** for assessing the predictive efficiency of the nomogram model. **(D,E)** ROC analysis of the 5-gene-based RF model based on 5-fold cross-validation in GSE5281 **(D)** and GSE122063 **(E)** datasets.

To further assess the predictive efficiency of the RF model, we first constructed a nomogram to estimate the risk of cuproptosis clusters in 310 AD patients (Figure 9A). The calibration curve and decision curve analysis (DCA) were applied for assessing the predictive efficiency of the nomogram model. According to the calibration curve, the error between the actual AD clusters risk and the predicted risk was very small (Figure 9B), and the DCA indicates that our nomogram has a high accuracy, which may provide a basis for clinical decision-making (Figure 9C). Subsequently, we validated our 5-gene prediction model on two external brain tissue datasets including normal subjects and AD patients. ROC curves showed satisfactory performance of the 5-gene prediction model with an AUC value of 0.8529 in the GSE5281 dataset and 0.8333 in the GSE122063 dataset (Figures 9D,E), indicating our diagnosis model is equally efficacious in distinguishing AD from normal individuals.

**Figure 10:**
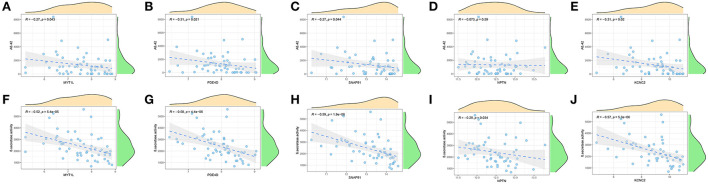
Validation of correlation analysis based on GSE106241 dataset. (A-E) Correlation between MYT1L **(A)**, PDE4D **(B)**, SNAP91 **(C)**, NPTN **(D)**, KCNC2 **(E)**, and Aβ-42 levels. **(F–J)** Correlation between MYT1L **(F)**, PDE4D **(G)**, SNAP91 **(H)**, NPTN **(I)**, KCNC2 **(J)**, and β-secretase activity.

Furthermore, we enrolled another external dataset (GSE106241) to validate the correlation between the predictor genes and biomarkers of AD that have been widely reported (Aβ-42 levels and β-secretase activity). We found that MYT1L, PED4D, SNAP91, and KCNC2 were negatively correlated with Aβ-42 levels (MYT1L, R = −0.27; PED4D, R = −0.31; SNAP91, R = −0.27; KCNC2, R = −0.31) and β-secretase activity (MYT1L, R = −0.52; PED4D, R = −0.58; SNAP91, R = −0.59; KCNC2, R = −0.57), while NPTN was only negatively associated with β-secretase activity (R = −0.29, Figures 10A–J). This result has proven the outstanding pathological diagnostic value of the 5-gene prediction model.

## Discussion

Due to the heterogeneity of AD pathology, the current treatment for AD lacks adequate efficacy (Lam et al., 2013; Byun et al., 2015). In the past decades, increasing advances have been made in anti-neurodegenerative therapy for AD, whereas the conventional classification based on histology allows for frequent drug resistance (Nandigam, 2008). Therefore, the identification of more appropriate molecular clusters is essential to guide the individualized treatment of AD. Cuproptosis, a recently reported form of copper-dependent cell death mainly evidenced by the aggregation of lipoylated mitochondrial enzymes, has been strongly implicated in the progression of the disease (Tang et al., 2022; Tsvetkov et al., 2022). However, the specific mechanisms of cuproptosis and its regulatory roles in various diseases have not been further investigated. Therefore, we attempted to elucidate the specific role of cuproptosis-related genes in AD phenotyping and the immune microenvironment. Additionally, gene signatures related to cuproptosis were utilized to predict the AD subtypes.

In this study, we comprehensively analyzed the expression profiles of cuproptosis regulators for the first time in brain tissues between normal subjects and AD patients. The dysregulated CRGs were found in AD patients more than those in normal individuals, suggesting a critical role of CRGs in the occurrence of AD. Subsequently, we calculated the correlation among CRGs to clarify the association between cuproptosis regulators and AD. We discovered that some cuproptosis modulators presented significant synergistic or antagonistic effects, which are evidenced by the existence of CRG interactions in AD patients. The abundance of immune cells were altered between control subjects and patients with AD. AD patients exhibited higher infiltration levels of B cells, T cells, neutrophils, resting NK cells, and macrophages, which were consensus with the previous studies verified in blood or brain tissues (Dey and Hankey Giblin, 2018; Dai and Shen, 2021; Paranjpe et al., 2021; Wang et al., 2022a). Further, we utilized unsupervised cluster analysis to illustrate the different cuproptosis regulation patterns in AD patients based on the expression landscapes of CRGs, and two distinct cuproptosis-related clusters were identified. Cluster2 exhibited elevated immune scores and relatively higher levels of immune infiltration. Cluster-specific DEGs indicated that Cluster1 was primarily enriched in mitochondrial localization, nerve growth, and development-related biological processes, while Cluster2 was characterized by immune cell activation and differentiation. It was reported that TGF-β signaling and Notch signaling were essential for the activation and differentiation of B cells and T cells (Cortez et al., 2016; Tsukumo and Yasutomo, 2018; Garis and Garrett-Sinha, 2020). Consistently, Cluster2 had a stronger activity of TGF-β signaling and Notch signaling. Taken together, it would be reasonable to believe that Cluster2 may possess more activated B cells and T cells to counteract the progression of AD and therefore showed a better prognosis.

In recent years, machine learning models based on demographic and imaging metrics have been increasingly applied for the prediction of AD prevalence (Falahati et al., 2014), and these studies confirm that multifactorial analyses have taken into account the relationships between variables, thus having a lower error rate and more reliable results compared to univariate analysis. In our current study, we compared the predictive performance of four selected machine learning classifiers (RF, SVM, GLM, and XGB) based on the expression profiles of cluster-specific DEGs and established an RF-based prediction model, which presented the highest predictive efficacy in the testing cohort (AUC = 0.9829), suggesting the RF-based machine learning has satisfactory performance in predicting the subtypes of AD. Subsequently, we selected five important variables (MYT1L, PDE4D, SNAP91, NPTN, and KCNC2) to construct a 5-gene-based RF model. MYT1L is a gene expressed exclusively in neurons that reprograms embryonic and infant fibroblasts into functional neurons and therefore acts as the critical regulator in the development of the nervous system, suggesting that MYT1L might be a potential treatment strategy for AD patients (Li et al., 2012). PDE4D is a member of the cAMP phosphodiesterase superfamily and is mainly involved in the regulation of cAMP activity. The role of PDE4D in AD is still controversial. It is reported that PDE4D inhibitors can improve cognitive function in the elderly and have mild gastrointestinal side effects, suggesting that PDE4D may be an effective therapeutic target for age-related neurodegenerative diseases (Bruno et al., 2011). However, another study demonstrated that the expression levels of PDE4D protein are negatively correlated with age and phosphorylated tau and positively correlated with the performance on frontal association cortex-related working memory tasks. Inhibition of PDE4D would make the frontal association cortex more vulnerable to degeneration (Leslie et al., 2020). Bioinformatics analysis indicated that SNAP91 could serve as a primary biomarker for Parkinson's disease and AD (Yemni et al., 2019; Hu et al., 2020). As a housekeeper of neuroplasticity, NPTN is closely associated with the regulation of synaptic plasticity, thus playing an important role in facilitating learning and memory. Studies have found that elevated KCNC2 might be implicated in the maturation of neuronal electrical activity during nervous system development, and a decrease in KCNC2 may aggravate the impairment of cognitive function in AD patients (Boda et al., 2012).

The 5-gene-based RF model can accurately predict AD in two external validation datasets (AUC = 0.8529 and 0.8333), which provides new insights into the diagnosis of AD. More importantly, we then constructed a nomogram model for the diagnosis of AD subtypes by using the MYT1L, PDE4D, SNAP91, NPTN, and KCNC2. We found this model exhibited remarkable predictive efficacy, indicating the value of this prediction model for clinical applications. Additionally, an increasing number of studies have confirmed that Aβ-42 levels and β-secretase activity are key pathological mechanisms contributing to the progression and poor prognosis of AD (Hampel et al., 2021; Cho et al., 2022). Therefore, we performed the correlation analysis between these five predictor genes with Aβ-42 levels and β-secretase activity in 60 AD samples from another external dataset. The results suggested that NPTN was negatively associated with β-secretase activity, while the other four predictor genes were negatively correlated with Aβ-42 levels and β-secretase activity. Taken together, the 5-gene-based RF model is a satisfactory indicator to assess AD subtypes and the pathological outcome of AD patients.

Some limitations need to be emphasized in this study. First, our current study was performed based on comprehensive bioinformatics analysis, and additional clinical or experimental assessment would be necessary to validate the expression levels of CRGs. Furthermore, more detailed clinical characteristics are required to confirm the performance of the prediction model. In addition, a greater number of AD samples are needed to clarify the accuracy of cuproptosis-related clusters and the potential correlation between CRGs and immune responses requires further exploration. Furthermore, though we applied an external dataset for additional validation, more experiments are necessary to prove the association between feature genes and Aβ-42 levels and β-secretase activity in AD pathology.

## Conclusion

In total, our study disclosed the correlation between CRGs and infiltrated immune cells and elucidated the significant heterogeneity of immune between AD patients with distinct cuproptosis clusters. A 5-gene-based RF model was selected as the optimal machine learning model, which can accurately assess AD subtypes and the pathological outcome of AD patients. Our study identifies for the first time the role of cuproptosis in AD and further elucidates the underlying molecular mechanisms leading to AD heterogeneity.

## Data availability statement

The datasets supporting the conclusions of this article are available in the GEO website (https://www.ncbi.nlm.nih.gov/geo/), with the following data accession identifiers: GSE33000, GSE5281, GSE122063, and GSE106241.

## Author contributions

YL designed the study, collected the original data, and drafted the initial manuscript. XL, CL, and LW searched related literature, finished the analysis, and visualized the final results. CL and YZ helped revise the manuscript. FL provided the funding and supervised the study. The final manuscript was read and approved by all authors. All authors contributed to the article and approved the submitted version.

## Funding

This work was supported by the High-level hospital foster grants from Fujian Provincial Hospital, Fujian province, China (Grant number: 2019HSJJ17), and the National Health and Family Planning Commission Grant (Grant number: WKJ-FJ-20).

## Conflict of interest

The authors declare that the research was conducted in the absence of any commercial or financial relationships that could be construed as a potential conflict of interest.

## Publisher's note

All claims expressed in this article are solely those of the authors and do not necessarily represent those of their affiliated organizations, or those of the publisher, the editors and the reviewers. Any product that may be evaluated in this article, or claim that may be made by its manufacturer, is not guaranteed or endorsed by the publisher.
